# Glucagon-like peptide-1 analogs against antipsychotic-induced weight gain: potential physiological benefits

**DOI:** 10.1186/1741-7015-10-92

**Published:** 2012-08-15

**Authors:** Bjørn H Ebdrup, Filip K Knop, Pelle L Ishøy, Egill Rostrup, Birgitte Fagerlund, Henrik Lublin, Birte Glenthøj

**Affiliations:** 1 Center for Neuropsychiatric Schizophrenia Research, CNSR and Center for Clinical Intervention and Neuropsychiatric Schizophrenia Research, CINS, Psychiatric Center Glostrup, University of Copenhagen, DK-2600 Glostrup, Denmark; 2 Diabetes Research Division, Department of Internal Medicine F, Gentofte Hospital, University of Copenhagen, DK-2900 Hellerup, Denmark; 3 Functional Imaging Unit, FIUnit, Glostrup Hospital, University of Copenhagen, DK-2600 Glostrup, Denmark

**Keywords:** Schizophrenia, antipsychotic medication, overweight, diabetes, GLP-1, exenatide, liraglutide, neuroprotection, cognition

## Abstract

**Background:**

Antipsychotic-induced weight gain constitutes a major unresolved clinical problem which may ultimately be associated with reducing life expectancy by 25 years. Overweight is associated with brain deterioration, cognitive decline and poor quality of life, factors which are already compromised in normal weight patients with schizophrenia.

Here we outline the current strategies against antipsychotic-induced weight gain, and we describe peripheral and cerebral effects of the gut hormone glucagon-like peptide-1 (GLP-1). Moreover, we account for similarities in brain changes between schizophrenia and overweight patients.

**Discussion:**

Current interventions against antipsychotic-induced weight gain do not facilitate a substantial and lasting weight loss. GLP-1 analogs used in the treatment of type 2 diabetes are associated with significant and sustained weight loss in overweight patients. Potential effects of treating schizophrenia patients with antipsychotic-induced weight gain with GLP-1 analogs are discussed.

**Conclusions:**

We propose that adjunctive treatment with GLP-1 analogs may constitute a new avenue to treat and prevent metabolic and cerebral deficiencies in schizophrenia patients with antipsychotic-induced weight gain. Clinical research to support this idea is highly warranted.

## Background

Schizophrenia is a severe and heterogeneous brain disease with a prevalence of approximately 0.5% in the world population [1]. Antipsychotics constitute the cornerstone in the treatment of schizophrenia. At the same time, much of the excess mortality in patients with schizophrenia may be attributed to metabolic side effects induced by antipsychotics [2]. The prevalence of overweight (body mass index (BMI) ≥25 kg/m^2^) and obesity (BMI ≥30 kg/m^2^) has been estimated at approximately 60% among schizophrenia patients [3] compared to less than 20 to 30% in the general population [4-7]. Accordingly, patients with schizophrenia may lose 25 or more years of life expectancy, with the majority of this excess in premature deaths being attributed to obesity-related cardiovascular disease and not to suicide [2,8].

Both genetic and environmental factors are contributing to this pronounced co-morbidity between schizophrenia and overweight [9,10]. Among the latter, antipsychotic drugs and, especially, second generation antipsychotics (SGAs) play a pivotal role [11,12]. Particularly, clozapine and olanzapine are associated with excessive weight gain (up to 2 kg per month) [13,14], but also the metabolism of glucose and lipids is affected to a varying degree [15,16]. Non-alcoholic fatty liver disease (NAFLD), which is a commonly known complication to obesity, may also be a possible side effect of antipsychotic drug treatment, and a high prevalence of NAFLD has been observed in schizophrenia patients [16]. Thus, in the present-day clinical setting optimal antipsychotic treatment is often compromised due to antipsychotic-induced weight gain and 'dysmetabolism'.

Obesity is considered the single most important risk factor for development of dyslipidaemia, diabetes, cardiovascular disease and ultimately premature death [17]. Also, obesity is associated with stigmatization and compromised quality of life [18,19]. According to the World Health Organization, schizophrenia is one of the seven most disabling diseases as measured in 'years lived with disability' among 15 to 44 year-olds, while diabetes is number 20 [20]. Co-morbidity between schizophrenia and obesity is likely to further impair quality of life and shorten the life span. Moreover, medical costs for obese individuals (without psychiatric co-morbidity) are 30% higher than those of normal weight individuals [21]. Accordingly, initiatives to promote a healthier weight will also have health economic benefits [22].

The following section begins by outlining current strategies against antipsychotic-induced weight gain, and by describing both peripheral and cerebral effects of the gut hormone glucagon-like peptide-1 (GLP-1). Next, we account for similarities in brain changes between schizophrenia [23] and overweight patients [24]. Finally, we propose that adjunctive treatment with a GLP-1 analog holds promise as a novel method to treat metabolic and cerebral deficiencies in schizophrenia patients with antipsychotic-induced weight gain.

## Discussion

### Current interventions against antipsychotic-induced weight gain

Current interventions against antipsychotic-induced weight gain fall into three categories. The first category involves changing antipsychotic medication to a compound less prone to result in weight gain. This strategy is recommended in clinical guidelines and results in a modest slowing of antipsychotic-induced weight gain to approximately 1.9 kg [11]. Change of medication, however, may increase the risk of exacerbating psychotic symptoms [11]. The second category involves lifestyle intervention and/or cognitive therapy and appears to slowdown the SGA-induced weight gain by 2.6 kg after 3 to 4 months; 4.2 kg after 6 months; and 3.1 kg after 12 to 18 months of treatment [25]. Finally, adjuvant medical treatment, the third category, has been attempted with numerous drugs, including ephedrine, sibutramine, orlistat, nizatidine, cimetidine, naltrexone, amantadine, reboxetine, fluoxetine, topiramate, dextroamphetamine, d-fenfluramine, famotidine, fluvoxamine, phenylpropanolamine, rosiglitazone, metformin and the combination of metformin and sibutramine [26]. Metformin is by far the most investigated of these drugs and is associated with the most pronounced deceleration of antipsychotic-induced weight gain: 2.9 kg over 13 weeks compared with placebo [26]. Metformin, however, is considered a weight neutral intervention in the treatment of type 2 diabetes [27].

Notably, all current interventions are weight neutral at best. This is the case because a weight reducing intervention may mask a net weight gain (for example, a reported weight loss of 4 kg may be based on a 2 kg weight gain in the intervention group compared with a 6 kg weight gain in the placebo group [28]). Absolute, placebo-controlled weight loss has only been achieved by means of resource-demanding interventions comprising strict diets and intensive physical training among hospitalized patients [29] or during short treatment periods (≤12 weeks) [30].

In the general population, treatment of obesity often comprises a combination of diet, exercise, modification of daily routines and medication [31]. Currently, orlistat (a lipase inhibitor), sibutramine (a serotonin-norepinephrine reuptake inhibitor) and sympathomimetics are the only approved drugs with the indication obesity [32]. Bariatric surgery (for example, gastric bypass surgery) is the single most efficient way to achieve lasting weight loss. However, this intervention is not without risk and, therefore, only indicated in selected patients (for example, morbid obesity (BMI ≥40 kg/m^2^)) [33].

Thus, there is an exigent need for better treatment strategies as well as a deeper understanding of the physiological consequences of the weight gain and the metabolic side effects associated with antipsychotic drugs. One such strategy may involve the effects of currently available GLP-1 analogs.

### GLP-1 physiology and GLP-1 based treatment

GLP-1 is a gut peptide hormone synthesized in endocrine L cells in the intestinal mucosa. GLP-1 is secreted into circulation after food intake. In the pancreas, GLP-1 stimulates glucose-induced insulin secretion (an incretin hormone) and inhibits glucagon secretion, thereby substantially contributing to maintaining the glucose homeostasis [34]. Activation of both peripheral GLP-1 receptors and GLP-1 receptors in the central nervous system reduces appetite and food intake thereby ensuring that body weight is kept down. In accordance with these observations, GLP-1 analogs for the treatment of type 2 diabetes have been developed (generally lowering HbA1c approximately 1%). A recent meta-analysis of studies of obese patients (with and without type 2 diabetes) demonstrated that ≥20 weeks of treatment with a GLP-1 analog (compared to other antidiabetic drugs, including metformin) induced a weight loss of 3 kg [35]. Since previous antidiabetic treatment strategies are generally associated with weight gain, the weight reducing effect of GLP-1 analogs may be of vital importance in the treatment of type 2 diabetes.

Clinically, the degree of weight loss appears to be positively correlated with the dose of GLP-1 analog. A recent two-year prospective study (including non-diabetic patients with baseline BMI ≥30 randomized to treatment with the GLP-1 analog liraglutide (2.4 to 3.0 mg once-daily) or placebo) reported a weight loss of 7.8 kg (compared to a 2 kg weight loss in the placebo group) [36]. Moreover, preclinical studies have indicated that GLP-1 analog treatment can reduce lipid accumulation in the liver [37,38].

Interestingly, a preclinical study has shown that olanzapine-induced weight gain and metabolic derangements in rats are reversed after treatment with the GLP-1 analog liraglutide [39].

### GLP-1 analogs

At present, six different GLP-1 analogs have been subject to trials: exenatide (Eli Lilly), liraglutide (Novo Nordisk), albiglutide (GlaxoSmithKline), taspoglutide (Ipsen and Roche), lixisenatide (Sanofi-Aventis) and LY2189265 (Eli Lilly). Only exenatide and liraglutide have been approved by the U.S. Food and the Drug Administration and European Medicines Agency. GLP-1 analogs have the indication type 2 diabetes in combination with metformin and/or sulphonylurea, when treatment with these drugs is insufficient. Much academic and commercial effort is being put into investigating the possibility of extending the indication to obesity. Both exenatide and liraglutide are administered subcutaneously. Exenatide exists in two formulations: twice daily (Byetta®) and once weekly (Bydureon®, Eli Lilly and Company, Indianapolis, Indiana, USA). Liraglutide (Victoza®, Novo Nordisk A/S, Bagsvaerd, Denmark) is administered once daily.

The major side effects of exenatide and liraglutide are mild to moderate nausea and vomiting. These side effects are dose-dependent and decline over time and they do not explain the observed weight loss associated with GLP-1 analog treatment [40]. The incidence of treatment-associated hypoglycemia is low (European Medicines Agency).

### Cerebral effects of GLP-1

Besides the effects of GLP-1 analogs on hyperglycemia and obesity (and possibly on hepatic lipid deposition), activation of central GLP-1 receptors may stimulate neural plasticity and prevent apoptosis, thereby suggesting involvement of GLP-1 in neuroprotection and learning [41,42]. An animal model of Alzheimer's disease has been used to show that the GLP-1 analog liraglutide prevents memory impairment and increases both neurogenesis as well as synaptic plasticity in the hippocampus [43,44]. In models of Parkinson's disease [45] and Huntington's chorea [46], dopaminergic neurons were protected and motor deficits improved when exposed to the GLP-1 analog exenatide.

### Structural brain changes in schizophrenia and overweight

It is well-established that schizophrenia is characterized by structural brain changes [47]. Already at first presentation of symptoms, reductions in total brain volume and hippocampus volume, along with increased ventricle volume, can be observed [48,49]. Moreover, the volumes of the dopamine-sensitive striatal regions appear to be reduced before initiation of antipsychotic treatment [50]. Progressive cerebral volume loss, for example, enlarged ventricles, is associated with more negative symptoms, cognitive decline and a more severe course of illness [23]. Current antipsychotic drugs have a limited effect on these symptom dimensions, and interventions with convincing neurotrophic properties are still lacking in the treatment of schizophrenia [51].

Growing evidence suggests that overweight is also associated with structural brain changes and cognitive deficits [24]. Large cross-sectional structural magnetic resonance imaging studies of obese patients have shown both global gray matter volume reductions (independently of age) [52] and widespread regional structural abnormalities in areas implicated in cognitive functions, impulse control and reward processing (for example, prefrontal cortex and striatum) [53,54]. These observations are supported by magnetic resonance spectroscopy and single-photon emission computed tomography studies suggesting that elevated BMI is associated with both neuronal abnormalities in frontal brain regions [55,56] and impaired cognitive function [57]. In schizophrenia, reduced prefrontal blood perfusion also appears to be associated with more negative symptoms [58]. Besides similarities in gray matter changes in schizophrenia and obesity, also coinciding white matter abnormalities in corpus callosum and fronto-temporal tracts have been reported [59,60].

Although brain abnormalities associated with obesity are already present in adolescents [61], it is still unclear if structural brain abnormalities precede the disorder as is the case in schizophrenia [62]. To our knowledge, weight loss has not been associated with changes in brain volume. In the available study addressing this issue, only global brain volume was assessed [63], thereby rendering the possibility that weight loss may still have a regional effect on brain volume. Nevertheless, some of the cognitive deficits associated with elevated BMI (that is, memory and attention/executive functioning) may be reversed after intentional weight loss [64].

### Functional brain changes in schizophrenia and overweight

Functional neuroimaging studies have further established the indications of a shared substrate for schizophrenia and obesity. For example, satiety appears to be controlled by the prefrontal cortex, which sends inhibitory inputs to the limbic and paralimbic regions, thereby suppressing hunger [65]. Studies using positron emission tomography have compared obese, previously obese and lean subjects and found abnormal cerebral blood flow in the insula and hippocampus in both obese and previously obese, but not in lean subjects [65]. This suggests that in obesity, disturbances in the satiety network are 'trait' rather than 'state' dependent. Interestingly, abnormal hippocampal-prefrontal connectivity appears to be present in first-episode schizophrenia, but also in subjects at high risk for developing schizophrenia [66].

More recently it has also been suggested that the regional disturbances in the responsivity of the reward system (for example, in the ventral striatum) may explain excessive food intake, thereby increasing the risk of developing overweight [67]. Of note, reward disturbances are also becoming increasingly recognized in schizophrenia patients as they even appear to precede the initiation of antipsychotic treatment [68].

### Potential clinical implications of GLP-1 analog treatment

First, encouraging and growing evidence supports that a sizable and enduring weight loss can be obtained by GLP-1 analog treatment in both diabetic and non-diabetic overweight or obese patients. Concurrently, an improved glycemic control is obtained. Second, a striking overlap appears to exist between key pathophysiological findings in schizophrenia and overweight/obesity. The extent to which this overlap is driven by the weight gain induced by antipsychotic medication or is caused by interactions from shared pathophysiological mechanisms warrants clarification. A shared substrate between schizophrenia and overweight will likely involve shared genetic risk factors [13]. With changes in body weight, physiological mechanisms may also come into play [64,69]. As such, exploration of the cerebral effects of GLP-1 analogs in overweight/obese subjects as well as in schizophrenia patients with antipsychotic induced weight gain will help to elucidate this proposed overlap between the two disorders.

An apparent way forward is initiation of clinical studies addressing whether GLP-1 analog treatment can reduce body weight in patients with antipsychotic-induced weight gain. In fact, there is currently a study investigating whether the GLP-1 analog exenatide can induce weight loss in obese patients treated with olanzapine [70]. Since previously un-medicated schizophrenia patients are in particularly high risk of developing metabolic side effects [71], addressing whether or not GLP-1 analog treatment can prevent weight gain is also pertinent. Finally, the indications of the procognitive and neurotrophic effects of GLP-1 call for further investigation. If the progressive brain loss and cognitive decline associated with schizophrenia and overweight/obesity can indeed be ameliorated by GLP-1 treatment, this will have major implications for future treatment of schizophrenia, but also for the treatment of obesity. In psychiatry, particular priority should be given to the effects of early adjunctive GLP-1 treatment, that is, in first-episode schizophrenia patients.

Current data certainly bear the connotation of adjunctive treatment with GLP-1 analog as a potential 'magic bullet' for addressing hitherto unresolved challenges in schizophrenia. Table 1 summarizes the hypothetical implications of GLP-1 treatment in antipsychotic-induced weight gain.

**Table 1:** Effects of GLP-1 analogs with relevance for schizophrenia

Unresolved challenges in schizophrenia	Effect of GLP-1 analog treatment	Current level of evidence	Reference(s)
**Endocrinological** **issues**			

Weight gain (non-diabetes)	Weight loss Improved glycaemic control (1% reduction in HbA1c) Reduction in plasma levels of cholesterol and liver enzymes Improved beta cell function Reduction in systolic and diastolic blood pressure	Prospective RCT* (2 years) and systematic review with meta-analyses of RCTs*	[35,36]

Diabetes (dysmetabolism)	Weight loss Improved glycaemic control (1% reduction in HbA1c) Reduction in plasma levels of cholesterol and liver enzymes Improved beta cell function Reduction in systolic and diastolic blood pressure	Cochrane systematic review and systematic review with meta-analyses of RCTs*	[35,72]

Non-alcoholic fatty liver disease	Reduction of hepatic lipid deposition	Preclinical studies	[37,38]

**Neuropsychiatric issues**			

Cognition	Improvement of memory deficits	Preclinical studies	[41,43]

Extrapyramidal side effects	Reduction of dyskinesia and regeneration of dopaminergic neurons	Preclinical studies	[45,46]

Neuroprotection	Increased neurogenesis and modulation of synaptic plasticity in the hippocampus	Preclinical studies	[43,44]

Quality of life	Improved well-being and reduced Hospital Anxiety and Depression Scale after six months compared to diabetic patients treated with insulin (the improvement was found to be independent of changes in body mass index)	Prospective, observational study	[73]

*RCT: Randomized clinical trial.

## Conclusions

Psychiatry is facing a major unmet need for addressing the severe metabolic side effects induced by antipsychotics. While awaiting the development of antipsychotic drugs with fewer side effects and more convincing neuroprotective and procognitive properties, an optimized treatment of schizophrenia may be achievable through a rational combination of drugs already registered, namely GLP-1 analogs. Current data converge towards adjunctive treatment with GLP-1 analogs as a potentially new avenue in the prevention and treatment of schizophrenia patients with antipsychotic-induced weight gain. Clinical research to support this idea is highly warranted.

## Abbreviations

BMI: body mass index; GLP-1: Glucagon-like peptide-1; NAFLD: Non-alcoholic fatty liver disease; SGAs: second generation antipsychotics

## Competing interests

BHE has received lecture fees from Eli Lilly and Company (producer of Byetta® and Bydureon®), and is on the Eli Lilly Danmark A/S Advisory Board. FKK has received lecture fees from Novo Nordisk A/S (producer of Victoza®) and Eli Lilly and Company and on Eli Lilly Danmark A/S Advisory Board. The other authors declare that they have no competing interests.

## Authors' contributions

BHE and FKK conceived of the paper and performed the literature search. BHE drafted the manuscript, which was then critically revised by each of the authors. Specifically, FKK evaluated the endocrinological studies; ER evaluated studies on magnetic resonance imaging and the implications for structural and functional brain changes; BF evaluated studies on cognition and the implications for potential cognitive improvement; and PLI, HL and BG evaluated the implications for metabolic disturbances in psychiatry. All authors read and approved the final manuscript.

## Pre-publication history

The pre-publication history for this paper can be accessed here:

http://www.biomedcentral.com/1741-7015/10/92/prepub
